# Embedding research in health systems: lessons from complexity theory

**DOI:** 10.1186/s12961-016-0128-x

**Published:** 2016-07-22

**Authors:** Louise Caffrey, Charles Wolfe, Christopher McKevitt

**Affiliations:** King’s College London, Division of Health and Social Care Research, Faculty of Life Sciences and Medicine, London, United Kingdom; Guy’s and St Thomas’ NHS Trust, London, United Kingdom; NIHR Biomedical Research Centre at King’s College London and Guy’s & St. Thomas’ NHS Trust, London, United Kingdom

**Keywords:** Health research systems, Implementation, Complexity theory, Organisational change, Systems approaches

## Abstract

**Background:**

Internationally, there has been increasing focus on creating health research systems. This article aims to investigate the challenges of implementing apparently simple strategies to support the development of a health research system. We focus on a case study of an English National Health Service Hospital Trust that sought to implement the national recommendation that health organisations should introduce a statement about research on all patient admission letters.

**Methods:**

We apply core concepts from complexity theory to the case study and undertake a documentary analysis of the email dialogue between staff involved in implementing this initiative.

**Results:**

The process of implementing a research statement in patient admission letters in one clinical service took 1 year and 21 days. The length of time needed was influenced firstly by adaptive self-organisation, underpinned by competing interests. Secondly, it was influenced by the relationship between systems, rather than simply being a product of issues within those systems. The relationship between the health system and the research system was weaker than might have been expected. Responsibilities were unclear, leading to confusion and delayed action.

**Conclusions:**

Conventional ways of thinking about organisations suggest that change happens when leaders and managers change the strategic vision, structure or procedures in an organisation and then persuade others to rationally implement the strategy. However, health research systems are complex adaptive systems characterised by high levels of unpredictability due to self-organisation and systemic interactions, which give rise to ‘emergent’ properties. We argue for the need to study how micro-processes of organisational dynamics may give rise to macro patterns of behaviour and strategic organisational direction and for the use of systems approaches to investigate the emergent properties of health research systems.

## Background

Globally, there has been increasing interest in creating health research systems, defined by WHO as “*the people, institutions, and activities whose primary purpose is to generate high quality knowledge that can be used to promote, restore and or maintain the health status of populations. It can include the mechanisms adopted to encourage the utilization of research*” [1]. Therefore, while scientists and physicians have long conducted health and medical research, a health research system refers to a systemic, coordinated approach to the generation and use of research in healthcare organisations. Health research systems can be conceptualised as existing at the intersection between the healthcare system and the research system and, as such, are a sub-set of the two [1]. A key question with regard to health research systems is how to organise them so as to achieve the system’s purpose. The journal *Health Research Policy and Systems* has led the call for ‘research on research’ in order to ensure that international learning on this question is based on empirical evidence rather than anecdote [2, 3].

In 2006, the English Department of Health set out its intention to “*create a health research system in which the NHS supports outstanding individuals, working in world-class facilities, conducting leading-edge research, focused on the needs of patients and the public*” [4]. The same year, the National Institute for Health Research (NIHR) was established in order to realise these goals and has continued to champion initiatives to support the development of the health research system. The United Kingdom (along with Canada) is recognised as a pioneer in the development of health research systems [3] and the NIHR recently described the infrastructure supporting health research in the United Kingdom as “*the most integrated health research system in the world*” [5].

In this article, we use core complexity theory concepts to analyse one NHS Hospital Trust’s experience of seeking to implement a national NIHR initiative aimed at supporting the integration of research in the health system. Complexity theory provides a conceptual framework for thinking about policy implementation and strategic change processes, which has increasingly been adopted in health service research [6–10]. Indeed, systems approaches, which the WHO suggests should be used to investigate health research systems [1], are built on the theoretical assumptions of complexity theory and are designed to address problems of organisational complexity [11]. We use this case study to illustrate how the implications of complexity can manifest in practice when we try to implement an initiative to support the integration of a research system and a health system. While this case study is situated in the English system, the findings are relevant to understanding implementation challenges in health research systems in general.

### Theoretical framework: complexity theory

Complexity theory provides a language and conceptual framework for thinking about the implementation of policy and the process of strategic change in complex adaptive systems [6, 8, 9, 12–17]. Any time we analyse an event or a process, whether consciously or not, we invoke a mental model, a way of thinking about the world. Instead of relying on implicit models, involving hidden assumptions and no clear framework to direct our analysis, complexity theory provides an explicit, established theoretical framework in which our assumptions are laid bare.

It has been suggested that complexity theory, as applied to organisations, echoes some of the ideas in the works of major sociologists [6], including Giddens [18], Hayek [19] and Schumpeter [20]. For the purpose of our analysis, the advantage of complexity theory is that it brings together disparate ideas and develops core concepts and a language to articulate them, in a systemic framework specifically designed to explain patterns in organisational change and policy implementation. Our analysis aims to provide a theoretically grounded illustration of how complexity can manifest when we try to implement initiatives to support health research systems.

Complexity theory suggests that health research systems are complex adaptive systems and that this would make implementing new initiatives unpredictable and therefore challenging. The theory spans a large and amorphous body of literature, which has been applied and developed across multiple disciplines. We focus on complexity theory as applied to organisational management [6, 8, 9, 12–17, 21, 22] and adopt three core complexity concepts, which have been used to explain the pattern of unpredictability in implementation processes. These concepts are systemic interactions, self-organisation and emergence; we outline these below. Several sources describe the properties of complex adaptive systems in more detail [6, 12–17].

### Systemic interactions

Complex organisations are characterised by systemic interactions [8, 15, 23, 24]. Unlike mechanical systems, for example, a car, which has a clear boundary, complex adaptive systems are open and so the boundaries are ‘fuzzy’. Agents (in the case of health research systems, people) in the system may be part of several other systems and membership of the systems is not fixed [8]. Indeed, as discussed above, health research systems necessarily involve dynamic interaction between research and health systems, such that the health research system can be seen as a sub-set of these two. As Pang et al. [1] suggest, the work of health research systems also necessitates interaction between disciplines in both systems. Further, in health research systems, the producers of research must interact with end-users, namely decision- and policymakers, health professionals, consumers (both public and private sectors), and the public [1]. Complexity theory asserts that the behaviour of the system will be difficult to predict, as it connects and interacts with other systems. Causal processes are non-linear [25]. Therefore, problem solving may be more difficult and the system may respond to change in unexpected ways [8]. This unpredictability will be compounded by the self-organising nature of the system, which is discussed below.

### Self-organisation

Complexity theory would further suggest that healthcare systems are adaptive and so self-organising, that is, they can organise themselves in the absence of external control, direction, pressure or influence [21, 26]. Complex systems are therefore constantly evolving. The trajectory of the system is influenced by structure – the relations between the parts of the system, which are a product of time and history. As Byrne puts it, “*The set of futures is path-dependent limited: not determined*”, it is “*bounded within a range*” [21]. Structures set the positions from which agents negotiate and structures influence the nature of negotiations.

However, complex systems are made up of individual agents with freedom to act in ways that are not always predictable. This unpredictability is compounded because the agents are connected and so the actions of each agent can change the context for others. Self-organisation is observed in non-human complex systems, for example, amongst termites [8] and flocks of birds as well as whole rainforests [27], but social systems are particularly complex because human beings have a high level of understanding and interpretative capacity and act on this basis. In this sense, the systemic interactions referred to above are also observed at the level of individual agents within systems. In other words, the agents in complex systems are themselves complex units within complex systems [28, 29].

Applying complexity theory to health research systems suggests that patients, clinicians, managers and others will act, react and adapt based on their individual perspectives and experiences [15, 21, 22]. There are almost always many possible reactions to any action and therefore the sum of these actions will be unpredictable. Sometimes, small changes will escalate into large outcomes. As noted above, structure creates an influence on the trajectory of complex systems. However, structure is also influenced by the daily decisions of agents who contribute to a constant creation and recreation of it [15, 22, 28]. This self-organising nature of complex systems implies that, while leaders and managers of health research systems can choose, plan and control the next intervention, they will not be able to choose, plan or control the outcomes of those interventions [15].

### Emergence

A key concept relating to both systemic interactions and self-organisation is emergence. According to complexity theory, interactions within the self-organising ‘whole’ of a system can produce ‘emergent’ properties that cannot be understood by examining each part in isolation [15, 21, 26]. Instead, these novel patterns arise at the macro-level from the dynamic interaction of micro-level parts and agents [26]. These emergent properties are unpredictable because they are not a product of the individual decisions of the people situated within parts of the system – the patterns are instead a property of the system.

A key tenet of complexity theory is its assertion that examining the micro-interactions of people is key to understanding the emergent properties of macro structures and the effects of policy change [15, 22, 23]. Moreover, examining micro-processes can highlight innovation as agents self-organise to create the new structures and behaviours needed to meet the demands of the relationships they have with each other and the environment [23, 26]. As Callaghan puts it, “*rather than ‘judging’ particular outcomes as dysfunctional we can understand the particular order that has been, and is being, negotiated*” [22]. Complexity theory’s aim is therefore to explain how things are, rather than to suggest how they should be. In this sense, it is a helpful theoretical foundation for seeking to understand the emergent properties of health research systems.

### Case study: introducing a reference to research in all patient admission letters

The case study focuses on the experience of one NHS Trust seeking to implement a particular initiative aimed at supporting the integration of research. The United Kingdom’s National Institute of Health Research (NIHR) has proposed a number of recommendations to, “*help* [health organisations] *to build a research culture*” [30]. One of the most seemingly straightforward of these is the suggestion to “*insert a standing research reference in all Trust patient admission letters*” [30]. The aim of this is to encourage patients to ask about research, thereby increasing the number of patients taking part in research and so increasing the research capacity of the NHS.

The case study Trust is part of an Academic Health Sciences Centre and hosts an NIHR Biomedical Research Centre (BRC), which aims to improve the translation of basic scientific developments into clinical benefits for patients and to reinforce the positon of the United Kingdom as a global leader in healthcare-related research. The Trust is ranked in the national top 10 trusts for both the quantity of its research and the number of patients recruited to clinical studies [31]. Nevertheless, the organisation prioritises continued improvement both of its ranking nationally (identified in published league tables [31]) and its levels of recruitment to studies, since income is attached to recruitment success [32]. Despite this, and to the frustration of those leading the process, the implementation of the research statement in outpatient appointment invitation letters in one clinical service (the pilot site), took 1 year and 21 days.

On October 3, 2013, our case study Trust’s Management Executive approved a proposal to insert a statement on all outpatient clinic letters, informing patients of the opportunity to participate in research. Prior to Trust-wide implementation, the statement was to be piloted and evaluated in one clinical area. The results below detail the process of implementing the statement in the pilot site as well as the process of negotiating where the statement would be piloted; as we discuss, the latter proved contentious. The research statement read as follows:

“*Our hospitals are involved in developing new treatments and better care. If you would like to take part in a research study, or want to know more about taking part, please speak to the doctor or nurse caring for you. If you are asked to take part in a research study, we will explain it to you in detail. If you decide not to take part, this will not affect your treatment in any way.*”

## Methods

Two of the authors (CM and CW) were involved in implementing the letter statement. LC, who was not involved, reviewed emails on the topic of implementing this change, which were sent and received by the BRC Manager leading the implementation process. In total, 40 staff were involved in the exchange of 90 emails.

These data were treated as documentary sources of information that had not been produced for the specific purpose of social research [33]. Scott [33] suggests that, in order to judge a source’s credibility, it is important to consider why it was created. In the case of our data, the emails have the advantage that, rather than being produced retrospectively for the purpose of research, they provide a contemporaneously produced account of the process that is framed and narrated by specific voices with specific agendas. In this sense, they have a high degree of credibility. They provide a window to observe the unfolding of the implementation process. The Trust R&D Department confirmed that ethical review was not required and approved the analysis.

LC analysed the emails thematically, using NVivo software to manage the data. CM subsequently reviewed the emails for validation. The analytic process drew heavily on Spencer et al.’s [34] ‘analytical hierarchy’, moving iteratively from descriptive coding through to themes, concepts and application of theory. Our analysis was presented to key actors involved in the process for discussion. There was agreement with the chronology of events we reconstructed, and with our analysis.

## Results

An overview of key events in the process of implementing the research statement in the pilot site is presented below in Table 1; a detailed exposition of the themes we identified follows.

**Table 1:** Overview of key events in the implementation of the pilot research statement

Dates	Action	Theme
2013		Making the decision for change
September	Patient and public advisory group and R&D Board approve proposal for change, each in one sitting	
3rd October	Trust Management Executive approves proposal	
3rd October–10th December	Discussion about research statement’s content: research only or research and education?	Self-organisation underpinned by conflict between groups
10th December	Board of Directors decides statement should exclusively concern research	
2014		
10th January	Board of Directors agree to pilot the statement in Services A and B	
1st April	Email from Trust General Manager with responsibility for patient letters to Service C (where trust review of patient letters already taking place) arranging to implement pilot statement about research and education in this service	
7th April	Biomedical Research Centre (BRC) Manager with responsibility for implementing research statement challenges General Manager that new wording is not what was agreed	
17th April	Trust Director for R&D decides to pilot research-only statement in a different service (Service D)	
30th April	Service D’s R&D Lead agrees to pilot statement in that service	Interaction between the health system and the research system
1st May	BRC Manager sends Service D’s Research Manager information about the letter statement	
25th June	BRC manager asks Service D’s R&D Lead to impress on the service’s Research Manager the importance of implementing the letter statement	
10th July	Research Manager says he will discuss with R&D Lead which clinics to implement the statement in and suggests the Manager for the wider clinical academic group may be responsible for implementation	
5th August	Following a request from the BRC Manager, the clinical academic group’s Deputy Manager contacts the R&D Lead offering to “discuss the roll out”	
18th August	R&D Lead emails BRC Manager to say no concerns reported from consultants “so please go ahead and arrange the necessary change in the clinic invite letter”	
28th August	R&D Lead emails again asking if new letters have been sent to patients; BRC manager replies that he thought R&D Lead would inform him when new letters had been sent; they identify that no one is implementing letter change	
29th August	R&D Lead asks clinical academic group’s Deputy Service Manger to liaise with administration to implement the change	
30th August	Clinical academic group’s Deputy Manager contacts her manager to ask him to implement research statement	
17th October	Clinical academic group’s Manager contacts Service D’s Service Manager and Patient Access Manager and asks them to change the letter	
24th October	Research statement is implemented in Service D	

### Factors affecting implementation

Our analysis identified a number of factors that did not significantly contribute to the length of time it took to implement the initiative. The official process for approving this initiative was relatively fast. The Trust Management Executive approved the proposal to insert the research statement in Trust outpatient admission letters in one sitting, as did the Research & Development (R&D) Board. The BRC’s Patient Public Involvement Advisory Group advised on the wording and approved this at one meeting. In 13 days, the R&D Lead (a senior clinician with responsibility for the conduct of research in the clinical service) in the pilot site gave permission for the pilot to take place. The final stage of the process, the editorial task of inserting the statement into the outpatient letter, was also relatively fast. Once the request was made to the relevant service manager and patient access manager, they contacted the relevant IT department responsible for managing outpatient letter templates, and within 1 week the statement had been added to the letter template. Therefore, roughly 1 year was taken up with other issues, which we now discuss.

#### Self-organisation

The initiative was affected by conflict between competing interests, which underpinned the properties of the system in a process of self-organisation. Two conflicting groups emerged in our analysis: the first group wanted a statement that exclusively concerned research. The second wanted the statement to also inform patients that they might be asked to support medical education by allowing access for medical learning. The group seeking a research-only statement comprised individuals with a top-level strategic or managerial responsibility for research within the Trust and the Biomedical Research Centre. It also included the Head of Trust Communications. This group argued that the statement would be most clear to patients if it exclusively concerned research. Those seeking to also include a statement about medical education were a small group of doctors in the Trust, with a responsibility for or particular interest in the education of medical students. They argued for the importance of efficiently informing patients about medical education and suggested that this change to Trust outpatient letters represented an opportunity in this regard.

From October 3 until December 10 there was negotiation between these groups about what the statement should include. On December 10 the Board of Directors (backed by the Head of Communications) decided that the statement should exclusively concern research in order to be clear to patients. On January 10, the Board of Directors agreed to pilot the statement in Service A and Service B.

However, the original proposal to the Trust Management Executive suggested that the letter change could take place alongside a Trust review of all outpatient letter templates, which was already underway. When the General Manager with responsibility for patient letters sought to implement the research statement, she did so, not in Service A or Service B, as had been decided by the Board of Directors, but in Service C, where the parallel review of all letter templates was due to start. Crucially, the Lead in this service was one of the doctors who had advocated for a statement about both research and education. This Lead and the General Manager sought approval for such a statement from an alternative source of authority within the Trust: the Trust Risk Quality Committee. The BRC Manager leading on implementation of the letter statement, challenged this move, arguing that the new wording was not what had been agreed by the Trust Management Executive. However, the General Manager and R&D Lead continued to seek approval for the alternative statement.

Faced with this, on April 17, after a total of 6 months and 2 weeks of negotiation, the Trust Director for R&D decided to pilot the research-only statement in a different clinical service. This effectively ended the negotiation between competing groups. The research statement would be implemented in Service D and, as reported above, gaining the service’s permission to implement was relatively fast (13 days).

#### Systemic interactions

From the point of permission being sought it took just over 6 months to implement the letter change in Service D. Our analysis suggests that the duration of time taken was affected by the interaction between the health system and the research system, rather than simply being influenced by issues within those systems themselves. The BRC Manager responsible for implementing the letter statement began by contacting Service D’s Research Manager. However, it seems the Research Manager did not act as he did not view implementing this initiative as part of his role. This was not explicitly stated but becomes clear through subsequent events. When, more than 3 weeks later, no action had been taken, the BRC Manager asked the R&D Lead to impress on the Research Manager the importance of implementing the statement. Meanwhile, the Research Manager agreed to speak with the R&D Lead about which outpatient clinics the letter should be implemented in but also suggested that the Manager for the wider clinical academic group within the trust that Service D sits within might be responsible for making the change, implying that the Research Manager did not view implementing this initiative as his role. Owing to a personal connection, the BRC Manager contacted the clinical academic group’s Deputy Manager who in turn contacted Service D’s R&D Lead offering to “*discuss the roll out*”, an ambiguous offer which did not make clear who was implementing the initiative.

On August 18, Service D’s R&D Lead emailed the BRC Manager to say that no concerns had been reported from the consultants, “*so please go ahead and arrange the necessary change in the clinic invite letter*”. On August 28, the R&D Lead emailed again asking if the new letters had been sent to patients and if the planned evaluation would be starting soon. The BRC manager replied that he thought the R&D Lead would inform him when the change had been implemented. The R&D Lead replied that he thought the manager was liaising with Service D’s administration team. Having identified that no action had been taken, the R&D Lead asked the clinical academic group’s Deputy Manager to liaise with administration to implement the change. The clinical academic group’s Deputy Manager contacted the clinical academic group’s Manager who directly asked Service D’s Service Manager and Patient Access Manager to change the letter. As outlined above, the letter statement was added 1 week later.

## Discussion

**Fig. 1:**
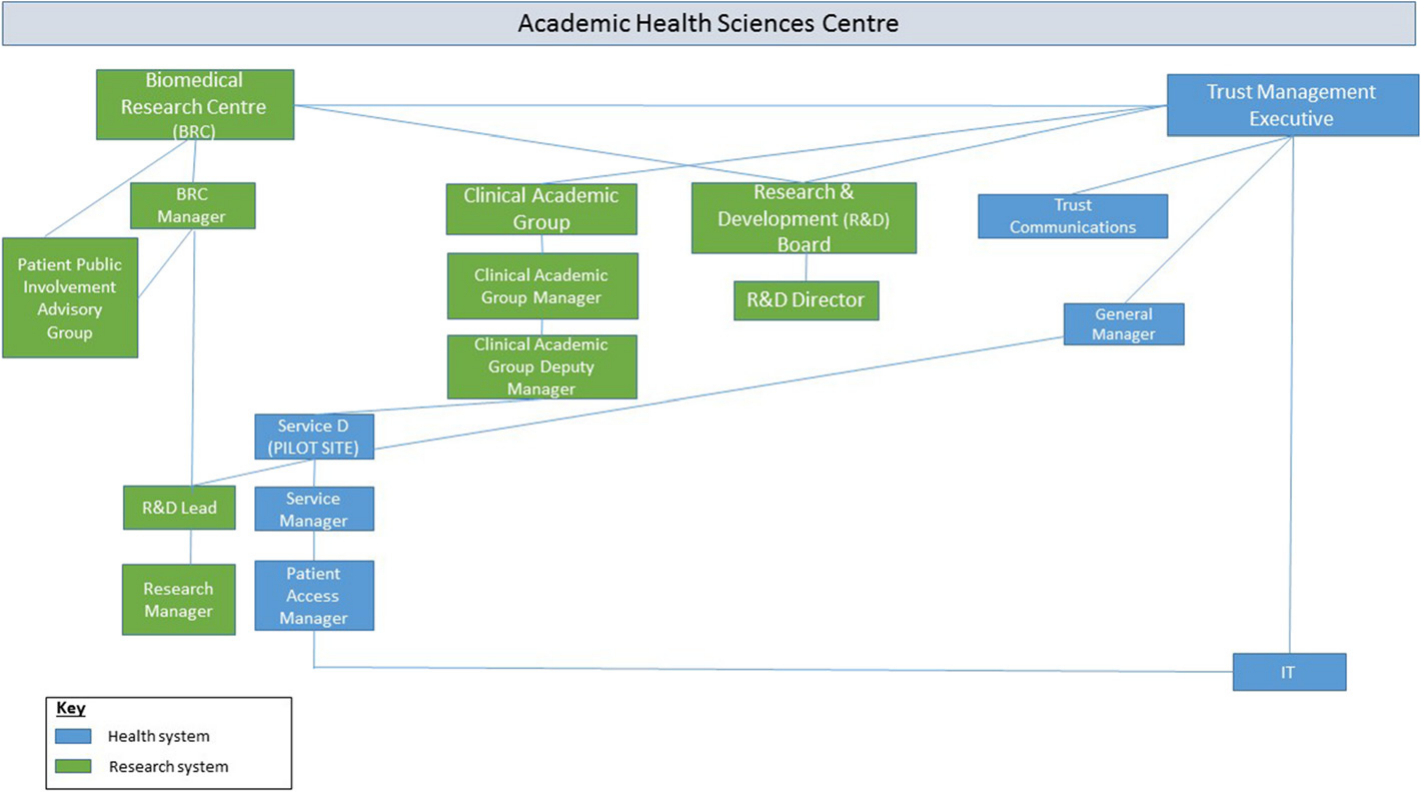
Conceptual model of health system and research system: components involved in implementing the research statement. Blue: Health system. Green: Research system

In keeping with the characteristics of complex adaptive systems, implementing this seemingly straight-forward initiative, to pilot a statement about research in Service D’s outpatient letters, involved two interacting systems – health and research – and a multitude of actors working within them. The statement needed to be approved by various bodies: the Trust Management Executive, the R&D Board, and the patient and public advisory group. Trust communications were also involved for advice on the wording. Its implementation was to be managed by a BRC Manager but also fell within the remit of the Trust General Manager with responsibility for patient letters. In seeking to implement the statement in Service D, the BRC Manager sought the permission of Service D’s R&D Lead. It seems he also contacted the R&D Lead and Research Manager in Service D with an expectation that they would implement this initiative. However, it was eventually decided that implementation fell within the remit of the Manager of the clinical academic group to which Service D belonged. Ultimately, the editorial task of changing the letter was undertaken by Service D’s Patient Access Manager and Service Manager, and by the Trust’s IT service. Figure 1 below provides an overview of the multiple parts of the health and research systems that, as illustrated above, were in practice, involved in implementing the letter statement, and the relationships between them.

Ultimately, the explanation for the delay in implementing the letter statement in Service D lies, not within research or within healthcare service delivery, but in the relationship between them. Responsibility for this initiative was located with a BRC Manager because, fundamentally, it was an initiative to support research. The BRC Manager sought to implement it by contacting research-focused staff, namely Service D’s R&D Lead and Research Manager. Indeed, he assumed that they would implement this change. Although aimed at supporting research, the change needed to be actioned by service staff (Service D’s Service Manager and Patient Access Manager) and it was not clear to actors how to action a clinical service change for the purpose of supporting research. Therefore, while the establishment of the BRC and Trusts’ membership of the Academic Health Science Centre might suggest strong relations between the health system and the health research system, our study highlights that the relationship was not strong enough to quickly implement this seemingly simple change. At the top, the necessary decisions by the Trust Management Executive and R&D Board were made with speed. However, amongst key actors on the ground, responsibility for action was unclear.

Our case study also illustrates self-organisation and its potential implications for implementing initiatives to support health research systems. As the failure to implement a research-only statement in Service C demonstrates, complex human systems are difficult, if not impossible, to control because the multiple actors within them each react and adapt based on their own knowledge and interpretation of the situation. In our case study, the objective of implementing the letter statement did not happen in a vacuum. Rather, it happened alongside other goals and initiatives – informing patients about the Trust’s medical education programme and the simultaneous Trust-wide review of letters. Actors each interpreted the situation from their own perspective and acted accordingly, with the result that two conflicting groups emerged and, through this process of self-organisation, the implementation process became unpredictable and protracted.

To borrow an analogy from biologist Richard Dawkins, complexity makes policy implementation in complex systems analogous to the difference between throwing a rock and throwing a live bird. While rocks and live birds are subject to the same laws of physics, with some basic information about the rock we can confidently predict where it will land; the same cannot be said of the bird [35]. Human systems are more like live birds than rocks and so decisions at the ‘top’ of a policy implementation hierarchy do not necessarily lead to expected effects [8, 12, 14, 36, 37]. In other words, the process of causality is non-linear. Given this, the emergent properties of the system are likely to depend, not just on the initiative that is being introduced, but on the interaction between the initiative and the (multiple) context(s) into which it is implemented [15, 28, 38]. Hence bad things sometimes happen to seemingly good policies [37].

Nonetheless, it is important to recognise that the systems did ultimately overcome obstacles to successfully implement the statement in Service D. In this respect, we observe how structural weaknesses, once recognised, can be addressed through a process of self-organisation, characterised by interactions between agents in systems. Indeed, this development underlies a key characteristic of complex systems: while the agency of actors may be perceived to frustrate top-down initiatives, their adaptive nature can also be a source of innovation [15, 22], problem-solving and learning [39], both amongst agents themselves and at a systemic level.

### Strengths and limitations

This study has a number of strengths and limitations. We use emails as documentary evidence to examine the implementation process but we were not able to undertake interviews with staff to explore their perspectives and behaviour in-depth. Two of the authors were, however, involved in the implementation process and so experienced it first hand and presented the findings to key actors. Further, the analysis of emails, rather than retrospective accounts, has the advantage that it provides a window to investigate the real-time unfolding of events and is therefore not influenced by potential interviewer effects.

Our analysis is limited to a single case study and so we cannot compare this implementation experience with those of other health organisations. However, it is important to note that it is not the case study that provides the abstracted theoretical principles we refer to; these come from complexity theory. Our purpose in applying these principles to a real-world case study is to demonstrate their practical utility for thinking about the challenge of embedding research in a health system.

## Conclusion

Health research systems are increasingly recognised as an important systemic mechanism, through which medical research can benefit population health. In illustrating how complexity can manifest when we try to introduce initiatives to support health research systems, we have sought to contribute to the growing body of literature that seeks to further our understanding of health research systems from an organisational perspective [1–3, 40].

Conventional ways of thinking about organisations suggest that change happens when leaders and managers change the strategic vision, structure or procedures in an organisation and then persuade others to rationally implement the strategy. However, as illustrated in our case study, complex adaptive systems, including health research systems, are self-organising and interactions throughout the whole of a system can produce emergent properties that are unpredictable. Indeed, in our case study, although there was no evidence that anyone was against the initiative to introduce a research statement per se, this did not mean that it could be easily implemented.

The properties of complex adaptive systems have implications for research on health research systems. Fundamentally, if complex adaptive systems are self-organising and demonstrate emergent properties emanating across whole systems, there is a need to focus not only on macro-structural changes to health research systems. Macro changes include policy changes, the establishment of institutional structures, like the United Kingdom’s NIHR and BRCs, as well as the quantitative outcomes of this, for example, levels of funding for research, the numbers of patients recruited to take part in medical research or the numbers of papers published. Instead, we need to also examine the micro-processes of organisational dynamics, namely how the connections and interactions between people give rise to macro patterns of behaviour and strategic organisational directions [15]. In other words, it is necessary to investigate the emergent behaviour of the interplay between structure and agency [22].

The application of complexity literature to health research systems suggests the specific importance of understanding how and whether health and research systems are being integrated at the micro level and what might support this transition and in what circumstances. As our case study illustrates, despite policy aimed at institutional integration (in this case through a BRC and an Academic Health Science Centre), the relationship between these systems may be weaker than expected in practice, and they may struggle to connect in certain circumstances. However, the findings also demonstrate the capacity of the system to innovate and for structural weaknesses to be addressed through actors’ agency [15, 39]. Therefore, there is a need for research to focus on understanding innovation in health research systems, potentially developing through the complex, self-organising interactions of individuals across whole systems, rather than necessarily through top-down processes.

Finally, it should be noted that, in this article, we have focused on illustrating the implications of complexity for implementation processes in health research systems. Systems approaches, which are based on the theoretical assumptions of complex adaptive systems [11], provide appropriate conceptual tools for investigating and deepening our understanding of the emergent properties of health research systems.
